# Pyridine-Based Heterocycles. Synthesis of New Pyrido [4',3':4,5]thieno[2,3-*d*]pyrimidines and Related Heterocycles

**DOI:** 10.3390/molecules15042651

**Published:** 2010-04-12

**Authors:** Hussein El-Kashef, Abdel-Rahman Farghaly, Ahmed Al-Hazmi, Thierry Terme, Patrice Vanelle

**Affiliations:** 1 Chemistry Department, Faculty of Science, Assiut University, 71516 Assiut, Egypt; 2 Chemistry Department, Faculty of Science, Ibb University, Yemen; 3 Laboratoire Pharmaco-Chimie Radicalaire, Faculté de Pharmacie, Universités d’Aix-Marseille I, II et III-CNRS, UMR 6264, Laboratoire Chimie Provence, 27 Bd J. Moulin, 13385 Marseille cedex 5, France

**Keywords:** pyrimidines, pyridines, thienopyrimidines, fused pyrimidines, fusedpyridines

## Abstract

The synthesis of the title compounds was achieved using ethyl 2-amino-6-methyl-4,5,6,7-tetrahydrothieno[2,3-*c*]pyridine-3-carboxylate (**1**) as starting material. The reaction of the amino ester **1** with phenylisothiocyanate in boiling ethanol afforded the thiourea derivative **5**. The cyclization reactions of **5** under different reaction conditions led to different pyridothienopyrimidine derivatives. Other reactions of the latter derivatives leading to pyrido[4',3':4,5]thieno[2,3-*d*]triazolo[1,5-*a*]pyrimidines are also presented.

## 1. Introduction

Fused pyridine ring systems have received considerable attention due to their diverse biological activity. In particular, pyrido[4',3':4,5]thieno[2,3-*d*]pyrimidin-4(3*H*)-ones were reported to be used for the prophylaxis and therapy of cerebral ischemia [1,2], and as central nervous system agents [3]. Other derivatives also act as 5-HT_1A_ receptor antagonists and serotonin reuptake inhibitors [4], β-lactamase inhibitors, analgesics, anti-inflammatory [5] and antimalarial agents [6]. Moreover, this triheterocyclic system showed highly potent dual 5-HT_1A_ and 5-HT_1B_ antagonists as potential antidepressant drugs [7]. The synthesis of other derivatives of this class of heterocycles were reported [8,9].

## 2. Results and Discussion

**Scheme 1:**
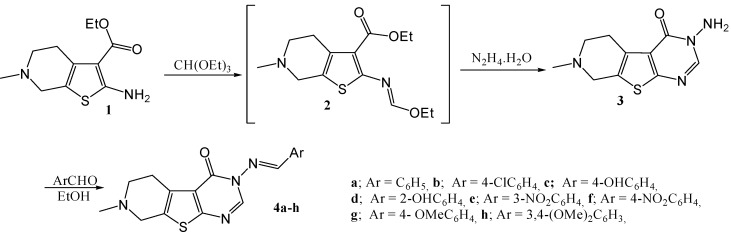
Synthesis of 3-arylideneamino-7-methyl-5,6,7,8-tetrahydro-3*H*-pyrido[4',3':4,5] thieno[2,3-*d*]pyrimidin-4-one (4a–h).

In view of the above findings and in continuation to our interest in the synthesis of polyfused heterocycles of biological importance [10,11,12,13,14,15,16,17,18,19,20,21,22,23], the synthesis of the title compounds was deemed of interest. Thus when the thienopyridine aminoester **1**, which is easily accessible via the Gewald reaction between 1-methylpiperidin-4-one, elemental sulfur and ethylcyanoacetate [7], was reacted with triethyl orthoformate, the corresponding ethoxymethyleneamino intermediate **2** was obtained as a reddish brown oil. This oil was reacted directly without any purification with hydrazine hydrate to give 3-amino-7-methyl-5,6,7,8-tetrahydro-3*H*-pyrido[4',3':4,5]thieno[2,3-*d*]pyrimidin-4-one (**3**). Conden-sation of **3** with aromatic aldehydes in boiling ethanol gave the corresponding arylidene derivatives **4a–h** (Scheme 1).

**Scheme 2:**
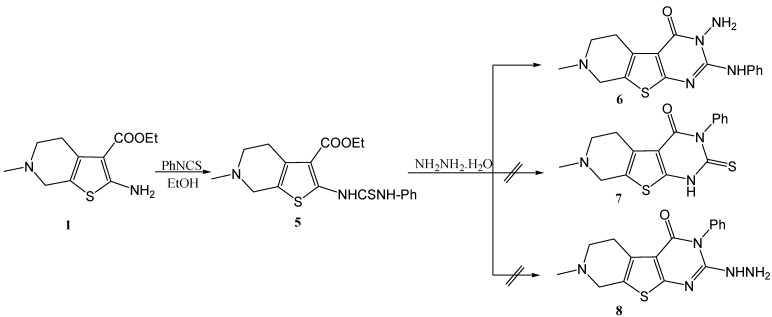
Synthesis of 3-amino-7-methyl-2-phenylamino-5,6,7,8-tetrahydro-3*H*-pyrido [4',3':4,5]thieno[2,3-*d*]pyrimidin-4-one (**6**) from the aminoester **1**.

The reaction of the amino ester **1** with phenylisothiocyanate in boiling absolute ethanol gave the corresponding thiourea derivative **5**. When **5** was allowed to react with hydrazine hydrate, the three possible structures **6** [24,25], **7** [5] and **8** [5] could be proposed for the reaction product (Scheme 2). Interestingly, in our hands this reaction gave one product only. The IR spectrum of this reaction product showed bands corresponding to primary NH_2_ functionality, thus from at first glance structure **7** could be ruled out.

**Scheme 3:**
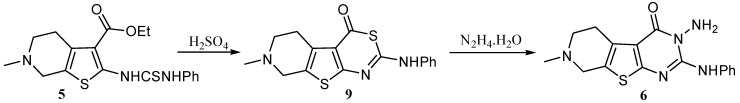
Synthesis of 3-amino-7-methyl-2-phenylamino-5,6,7,8-tetrahydro-3*H*-pyrido [4',3':4,5]thieno[2,3-*d*]pyrimidin-4-one (**6**) from the thioureido ester **5**.

An unequivocal synthesis was carried out to prove whether **6** or **8** is the structure of the reaction product. Thus compound **5** was treated with concentrated sulfuric acid it gave the thiazinone derivative **9** which was then reacted with hydrazine hydrate, whereby an extrusion of the sulfur atom with its replacement by an *N*-amino nitrogen atom occurred, giving 3-amino-7-methyl-2-phenylamino-5,6,7,8-tetrahydro-3*H*-pyrido[4',3':4,5]thieno[2,3-*d*]pyrimidin-4-one (**6**) having the same melting point, mixed melting point and spectral data as those of our reaction product (Scheme 3).

**Scheme 4:**
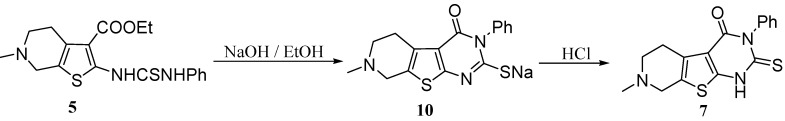
Synthesis of the pyridothienopyrimidinonethione **7** from **5**.

On the other hand, compounds **7** and **8** could be obtained following different reaction pathways *via* the procedure reported by El-Baih *et al.* [26]. Thus, **7 **could be obtained when the thiourea derivative **5** was heated under reflux in ethanolic sodium hydroxide giving the sodium salt **10** which gave **7** upon acidification with hydrochloric acid (Scheme 4).

**Scheme 5:**
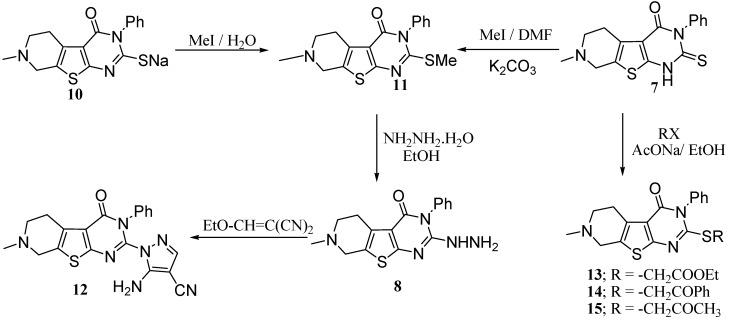
Synthesis of different pyridothienopyrimidines.

Compound **8** was easily obtained by treatment of the 2-methylthio derivative **11** with hydrazine hydrate in boiling ethanol. It is to be mentioned that compound **11** was obtained by methylation of either the thione **7** in DMF, or by methylation of the sodium thiolate 10 in an aqueous solution as shown in Scheme 5. Other alkylations of the thione **7** with ethyl chloroacetate, phenacyl bromide and chloroacetone gave the corresponding products **13**–**15** respectively. When **8** was allowed to react with ethoxymethylene malononitrile the reaction product was identified as 5-amino-1-(7-methyl-3-phenyl-5,6,7,8-tetrahydro-3*H*-pyrido[4',3':4,5]thieno[2,3-*d*]pyrimidin-4-on-2-yl)-1*H*-pyrazole-4-carbonitrile **12**) (Scheme 5).

**Scheme 6:**
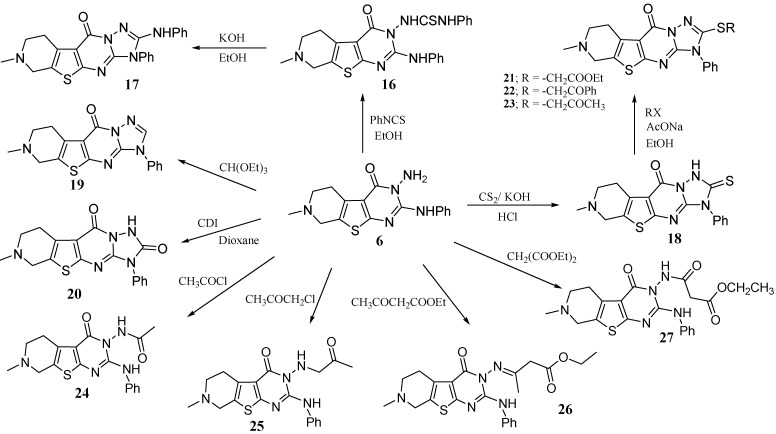
Pyridothienopyrimidine **6** as a versatile key intermediate.

Compound **6** proved to be a key intermediate and served for the preparation of several new fused heterocycles related to pyridothienopyrimidine ring system (Scheme 6). 

Thus the triazolo derivative **17** could be obtained via the reaction of **6** with phenylisothiocyanate in boiling ethanol to give the thiourea derivative **16**, which underwent a cyclodehydrosulfurization when heated in ethanolic KOH under reflux to afford 7-methyl-2-phenylamino-3-phenyl-6,7,8,9-tetrahydro-3*H*-pyrido[4',3':4,5]thieno[2,3-*d*][1,2,4]triazolo[1,5-*a*]pyrimidin-10-one (**17**). Other triazolo derivatives could be obtained by the reaction of compound **6 **with carbon disulfide, triethyl orthoformate and 1',1'-carbonyldiimidazole (CDI), giving compounds **18–20** respectively. The thione **18** was alkylated with ethyl chloroacetate, phenacyl bromide and chloroacetone in boiling ethanol in the presence sodium acetate to give the corresponding products **21-23** respectively. Compound **6**, when treated with acetyl chloride also gave *N*-(7-methyl-2-phenylamino-5,6,7,8-tetrahydro-3*H*-pyrido[4',3':4,5]thieno[2,3-*d*]pyrimidin-4-on-3-yl)acetamide (**24**). The reaction of **6** with chloroacetone in boiling ethanol afforded the corresponding 7-methyl-2-phenylamino-3-(2-oxopropylamino)-5,6,7,8-tetrahydro-3*H*-pyrido- [4',3':4,5]thieno[2,3-*d*]pyrimidin-4-one (**25**). When **6 **was reacted with ethyl acetoacetate**, **a condensation reaction took place only between the keto carbonyl group of the ketoester and the primary amino group of compound **6** giving ethyl 3-(7-methyl-2-phenylamino-5,6,7,8-tetahydro-3*H*-pyrido[4',3':4,5]thieno[2,3-*d*]pyrimidin-4-on-3-ylimino)butanoate (**26**), whereas, the reaction of **6** with diethyl malonate gave ethyl 3-(7-methyl-2-phenylamino-5,6,7,8-tetahydro-3*H*-pyrido[4',3':4,5]thieno [2,3-*d*]pyrimidin-4-on-3-ylamino)-3-oxopropanoate (**27**) (Scheme 6). 

**Scheme 7:**
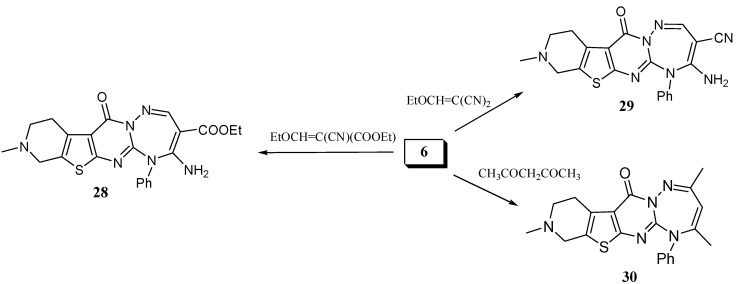
Synthesis of pyridothienopyrimidotriazepines from **6**.

It is worth mentioning that the reaction of **6 **with ethyl ethoxymethylene cyanoacetate and ethoxymethylenemalononitrile gave the triazepines **28 **and **29 **respectively (Scheme 7). Another triazepine derivative was obtained when **6 **was reacted with acetylacetone in boiling ethanol to give 5-phenyl-2,4,9-trimethyl-8,9,10,11-tetrahydro-5*H*-pyrido[4'',3'':4',5']thieno[2',3':4,5]pyrimido[1,2-*b*][1,2,4]-tri-azepin-12-one (**30**) (Scheme 7). 

## 3. Experimental

### 3.1. General

All melting points are uncorrected and were measured on a Gallankamp melting point apparatus. The IR spectra were recorded on a Shimadzu 470 IR-Spectrophotometer using the KBr wafer technique. The ^1^H-NMR spectra were measured on a Jeol LA 400 MHz FT-NMR, Varian Unity-plus 300 (200 MHz) or Varian EM-390 (90 MHz) spectrometers with TMS as internal standard (δ values in ppm). Elemental analyses were carried out using a Perkin-Elmer 240C Microanalyzer, and the results were within an acceptable range.

*3-Amino-7-methyl-5,6,7,8-tetrahydro-3H-pyrido[4',3':4,5]thieno[2,3-d]pyrimidin-4-one* (**3**). A mixture of **1** (0.480 g, 2 mmol) and triethyl orthoformate (5 mL) was heated under reflux for 6 h. After cooling, the solvent was removed *in vacuo* and the brownish red oil obtained was used directly without purification in the next step. A mixture of the latter intermediate (0.592 g, 2 mmol) and excess of hydrazine hydrate (5 mL) in ethanol (10 mL) was refluxed for 1 h. The solvent was then removed under reduced pressure. The solid product obtained was triturated with water, filtered and recrystallized from ethanol as pale buff crystals, yield 0.48 g (81%), m.p. 194–196 ºC. IR: υ cm^-1^ = 3,450, 3,300 (NH_2_), 2,910 (CH alph.), 1,670 (C=O).^1^H-NMR (CDCl_3_): δ = 2.50 (s, 3H, NCH_3_), 2.80 (m, 2H, CH_2_), 3.20 (m, 2H, CH_2_), 3.67 (s, 2H, CH_2_), 5.13 (bs, 2H, NH_2_), 8.20 (s, 1H, CH pyrimidine). C_10_H_12_N_4_OS (236.30) Calcd.: C, 50.83; H, 5.12;N, 23.71; S, 13.57. Found: C, 50.93; H, 4.92; N, 23.41; S, 13.87.

### 3.2. General procedure for the synthesis of 3-arylideneamino-7-methyl-5,6,7,8-tetrahydro-3H-pyrido[4',3':4,5]thieno[2,3-*d*]pyrimidin-4-ones **4a–h**

A mixture of compound **3 **(0.236 g, 1 mmol) and the appropriate aromatic aldehyde (0.001 mol) in ethanol (10 mL) was refluxed for 2 h. After cooling, the solid precipitate was collected and recrystalized from appropriate solvent to give **4a–h**.

*3-(Benzylideneamino)-7-methyl-5,6,7,8-tetrahydro-3H-pyrido[4',3':4,5]thieno[2,3-d]pyrimidin-4-one* (**4a**). This compound was obtained from ethanol as yellow crystals, yield 0.30 g (92%), m.p. 166–168 ºC. IR: υ cm^-1^ = 3,050 (CH arom.), 2,920 (CH aliph.), 1,670 (C=O). ^1^H-NMR (CDCl_3_): δ = 2.43 (s, 3H, NCH_3_), 2.67 (t, 2H, CH_2_), 3.10 (t, 2H, CH_2_), 3.80 (s, 2H, CH_2_), 6.83 (m, 3H, Ar-H),7.76 (m, 2H, Ar-H), 8.10 (s, 1H CH, pyrimidinone), 9.20 (s, 1H, N=CH). C_17_H_16_N_4 _OS (324.40) Calcd.: C, 62.94; H, 4.97; N, 17.27; S, 9.88. Found: C, 62.72; H, 4.82; N, 16.94; S, 9.95.

*3-(4-Chlorobenzylideneamino)-7-methyl-5,6,7,8-tetrahydro-3H-pyrido[4',3':4,5]thieno[2,3-d]pyrimidin-4-one* (**4b**). This compound was obtained from ethanol as pale yellow crystals, yield 0.21 g (85%), m.p. 198–200 ºC. IR: υ cm^-1 ^= 3,030 (CH arom.), 2,950 (CH aliph.), 1,675 (C=O).^ 1^H-NMR (CDCl_3_): δ = 2.43 (s, 3H, NCH_3_), 2.70 (t, 2H, CH_2_), 3.07 (t, 2H, CH_2_), 3.56 (s, 2H, CH_2_), 7.36 (d, 2H, *J* = 8 Hz, Ar-H), 7.69 (d, 2H, *J* = 8 Hz, Ar-H), 8.14 (s, 1H, CH pyrimidinone), 9.54 (s, 1H, N=CH). C_17_H_15_ClN_4_OS (358.85) Calcd.: C, 56.90; H, 4.21; Cl, 9.88; N, 15.61; S, 8.94. Found: C, 56.68; H, 4.03; Cl, 9.38; N, 15.30; S, 8.66.

*3-(4-Hydroxybenzylideneamino)-7-methyl-5,6,7,8-tetrahydro-3H-pyrido[4',3':4,5]thieno[2,3-d]pyrimidin-4-one* (**4c**). This compound was obtained from ethanol as yellow crystals, yield 0.21 g (62%), m.p. 224–226 ºC. IR: υ cm^-1 ^= 3,350 (OH), 3,050 (CH arom.), 2,900 (CH aliph.), 1,670 (C=O). ^1^H-NMR (CDCl_3_): δ = 2.32 (s, 3H, NCH_3_), 2.61 (t, 2H, CH_2_), 2.93 (t, 2H, CH_2_), 3.49 (s, 2H, CH_2_), 6.89 (d, 2H, *J* = 8.5 Hz, Ar-H), 7.72 (d, 2H, *J* = 8.5 Hz, Ar-H), 8.37 (s, 1H, CH pyrimidinone), 8.87 (s, 1H, N=CH), 11.02 (s, 1H, OH). C_17_H_16_N_4_O_2_S (340.41) Calcd.: C, 59.98; H, 4.74; N, 16.46; S, 9.42. Found: C, 59.68; H, 4.55; N, 16.16; S, 9.23.

*3-(2-Hydroxybenzylideneamino)-7-methyl-5,6,7,8-tetrahydro-3H-pyrido[4',3':4,5]thieno[2,3-d]pyrimidin-4-one* (**4d**). This compound was obtained as white powder from ethanol, yield: 0.29 g (86%), m.p. 200–202 ºC. IR: υ cm^-1 ^= 3,320 (OH), 3,090 (CH arom.), 2,930 (CH aliph.), 1,670 (C=O). ^1^H-NMR (CDCl_3_): δ = 2.34 (s, 3H, NCH_3_), 2.93 (t, 2H, CH_2_), 3.17 (t, 2H, CH_2_), 3.67 (s, 2H, CH_2_), 7.33 (m, 4H, Ar-H), 8.21 (s, 1H, CH pyrimidine), 9.45 (s, 1H, N=CH), 10.90 (s, 1H, OH). C_17_H_16_N_4_O_2_S (340.40)Calcd.: C, 59.98; H, 4.74; N, 16.46; S, 9.42. Found: C, 59.66; H, 4.49; N, 16.69; S, 9.81.

*3-(3-Nitrobenzylideneamino)-7-methyl-5,6,7,8-tetrahydro-3H-pyrido[4',3':4,5]thieno[2,3-d]pyrimidin-4-one* (**4e**). This compound was obtained from ethanol as yellow crystals, yield: 0.30 g, (81%), m.p. 208–210 ºC. IR: υ cm^-1 ^= 3,020 (CH arom.), 2,960 (CH aliph.), 1,675 (C=O), 1,520, 1,350 (NO_2_). ^1^H-NMR (DMSO-d_6_): δ = 2.47 (s, 3H, NCH_3_), 2.77 (t, 2H, CH_2_), 3.13 (t, 2H, CH_2_), 3.62 (s, 2H, CH_2_), 7.61 (m, 1H, Ar-H), 8.16 (m, 1H, Ar-H), 8.30 (m, 1H, Ar-H), 8.36 (s, 1H, Ar-H), 8.60 (s, 1H, CH pyrimidine), 9.83 (s, 1H, N=CH). C_17_H_15_N_5_O_3_S (369.40) Calcd.: C, 55.27; H, 4.09; N, 18.96; S, 8.68. Found: C, 54.98; H, 3.81; N, 18.88; S, 8.95.

*3-(4-Nitrobenzylideneamino)-7-methyl-5,6,7,8-tetrahydro-3H-pyrido[4',3':4,5]thieno[2,3-d]pyrimidin-4-one* (**4f**). This compound was obtained from ethanol as yellowish crystals, yield 0.34 g (92%), m.p. 218–220 ºC. IR: υ cm^-1 ^= 3,020 (CH arom.), 2,950 (CH aliph.), 1,670 (C=O), 1,530, 1,330 (NO_2_). ^1^H-NMR (CDCl_3_): δ = 2.91 (s, 3H, NCH_3_), 3.18 (t, 2H, CH_2_), 3.44 (t, 2H, CH_2_), 4.39 (s, 2H, CH_2_),8.27 (m, 3H, Ar-H + CH pyrimidinone), 8.37 (d, 2H, *J* = 8.8 Hz, Ar-H), 8.79 (s, 1H, N=CH). C_17_H_15_N_5_O_3_S (369.40) Calcd.: C, 55.27; H, 4.09; N, 18.96; S, 8.68. Found: C, 54.99; H, 3.81; N, 18.18; S, 7.95. 

*3-(4-Methoxybenzylideneamino)-7-methyl-5,6,7,8-tetrahydro-3H-pyrido[4',3':4,5]thieno[2,3-d]pyrimidin-4-one* (**4g**). This compound was obtained from ethanol as yellowish crystals, yield 0.21 g (59%), m.p. 168–170 ºC. IR: υ cm^-1^ = 3,050 (CH arom.), 2,900 (CH aliph.), 1,665 (C=O). 1H-NMR (CDCl_3_): δ = 2.43 (s, 3H, NCH_3_), 2.70 (t, 2H, CH_2_), 3.09 (t, 2H, CH_2_), 3.56 (s, 2H, CH_2_), 3.79 (s, 3H, OCH_3_), 6.89 (d, 2H, *J* = 8.7 Hz, Ar-H), 7.71 (d, 2H, *J* = 8.7 Hz, Ar-H), 8.13 (s, 1H, CH pyrimidinone), 9.24 (s, 1H, N=CH). C_18_H_18_N_4_O_2_S (354.43) Calcd.: C, 61.00; H, 5.12; N, 15.81; S, 9.05. Found: C, 60.15; H, 4.31; N, 15.55; S, 8.85.

*3-(3,4-Dimethoxybenzylideneamino)-7-methyl-5,6,7,8-tetrahydro-3H-pyrido[4',3':4,5]thieno[2,3-d]pyrimidin-4-one* (**4h**). This compound was obtained from ethanol as yellow crystals yield 0.22 g (57%), m.p. 162–164 ºC. IR: υ cm^-1^ = 3,090 (CH arom.), 2,940 (CH aliph.), 1,665 (C=O).^1^H-NMR (CDCl_3_): δ = 2.47 (s, 3H, NCH_3_), 2.74 (t, 2H, CH_2_), 3.13 (t, 2H, CH_2_), 3.61 (s, 2H, CH_2_), 3.91 (s, 3H, OCH_3_), 3.92 (s, 3H, OCH_3_), 6,99 (d, 1H, *J* = 8.5 Hz, Ar-H), 7.24 (dd, 1H, *J* = 2.5, 8.5 Hz, Ar-H), 7.36 (d, 1H *J* = 2.5, Ar-H), 8.19 (s, 1H, CH pyrimidinone), 9.23 (s, 1H, N=CH). C_19_H_20_N_4_O_3_S (384.45) Calcd.: C, 59.36; H, 5.24; N, 14.57; S, 8.34. Found: C, 59.05; H, 4.94; N, 14.85; S, 8.30.

*Ethyl 6-methyl-2-(3-phenylthioureido)-4,5,6,7-tetrahydrothieno[2,3-c]pyridine-3-carboxylate* (**5**). A mixture of **1** (7.2 g, 30 mmol) and phenylisothiocyanate (4.1 g, 30 mmol) in absolute ethanol (20 mL) was heated under reflux for 2 h. After cooling, the solid product was collected, dried and recrystalized from ethanol to give yellow crystals, yield 7.01 g (62%), m.p. 201–202 ºC. IR: υ cm^-1 ^= 3,180 (NH), 2,900 (CH aliph.), 1,670 (C=O), 1,190 (C=S). ^1^H-NMR (DMSO-d_6_): δ = 1.24 (t, 3H, *J* = 7.1 Hz, CH_2_CH_3_), 2.31 (s, 3H, NCH_3_), 2.57 (t, 2H, CH_2_), 2.75 (t, 2H, CH_2_), 3.54 (s, 2H, CH_2_), 4.19 (q, 2H, *J* = 7.1 Hz, CH_2_CH_3_), 7.21 (m, 3H, Ar-H), 7.38 (m, 2H, Ar-H), 8.45 (s, 1H, NH), 12.07 (s, 1H, NH). C_18_H_21_N_3_O_2_S_2_ (375.51) Calcd.: C, 57.57; H, 5.64; N, 11.19; S, 17.08. Found: C, 57.33; H, 5.36; N, 10.96; S, 16.80.

*3-Amino-7-methyl-2-phenylamino-5,6,7,8-tetrahydro-3H-pyrido[4',3':4,5]thieno[2,3-d]pyrimidin-4-one* (**6**): A mixture of the arylthiourea **5** (3.75 g, 10 mmol) and excess hydrazine hydrate (5 mL) was heated under reflux for 12 h. After cooling, the precipitate formed was filtered, dried and recrystalized from ethanol/dioxane (1:2) to give white crystals, yield: 2.03 g (62%), m.p. 252–254 ºC. IR: υ cm^-1 ^= 3,350, 3,300 (NH_2_), 3,200 (NH), 3,050 (CH arom.), 2,900 (CH aliph), 1,660 (C=O). ^1^H-NMR (DMSO-d_6_): δ = 2.41 (s, 3H, NCH_3_), 2.64 (t, 2H, CH_2_), 2.98 (t, 2H, CH_2_), 3.40 (s, 2H, CH_2_), 4.79 (bs, 2H, NH_2_), 7.32 (m, 2H, Ar-H), 7.61 (m, 3H, Ar-H), 8.51 (bs, 1H, NH). MS: *m/z* (rel. int.) 329 (M^+^, 91%), 328 (40), 327 (100), 253 (22.8), 77 (20). C_16_H_17_N_5_OS (327.40) Calcd.: C, 58.69; H, 5.23; N, 21.39; S, 9.79. Found: C, 58.59; H, 4.95; N, 21.11; S, 9.89. 

*7-Methyl-3-phenyl-2-thioxo-1,2,5,6,7,8-hexahydro-3H-pyrido[4',3':4,5]thieno[2,3-d]pyrimidin-4-one* (**7**). *Method (a):* A suspension of compound **5** (3.75 g, 10 mmol) in an ethanolic solution of sodium hydroxide (2*N*, 5 mL) was heated under reflux for 2 h. The reaction mixture was cooled and acidified with hydrochloric acid (2*N*), whereupon a solid product was separated out. The solid product formed was filtered and recrystallized from ethanol as yellow crystals, yield 3.1 g (94%), m.p. 296–298 ºC. IR: υ cm^-1 ^= 2900 (CH aliph.), 2400 (SH), 1680 (C=O). ^1^H-NMR (DMSO-d_6_): δ = 2.33 (s, 3H, NCH_3_), 2.83 (m, 4H, 2CH_2_), 3.33 (s, 2H, CH_2_), 7.13 (m, 3H, Ar-H), 7.33 (m, 2H, Ar-H). MS : *m/z* (rel. int.) 329 (M^-2^, 57%), 324.35 (13.5), 299.22 (24.8), 285.25 (41.9), 252.70 (24.5), 76.83 (33), 17.96 (24.4). C_16_H_15_N_3_OS_2_ (329.44) Calcd.: C, 58.33; H, 4.59; N, 12.75; S, 19.47. Found: C, 58.45; H, 4.35; N, 12.88; S, 19.25. *Method (b):* An aqueous solution of compound **10 **(3.83 g, 10 mmol) was acidified with concentrated hydrochloric acid whereupon a solid product was separated out which was filtered, washed with water and recrystallized from ethanol to give as yellow crystals, yield: 3.28 g (86%). 

*2-Hydrazino-7-methyl-3-phenyl-5,6,7,8-tetrahydro-3H-pyrido[4',3':4,5]thieno[2,3-d]pyrimidin-4-one* (**8**). A mixture of **11 **(0.37 g, 1 mmol) and hydrazine hydrate (5 mL) in ethanol (10 mL) was heated under reflux for 2 h. The reaction mixture was then allowed to cool and the solid product obtained was collected and recrystallized from ethanol as white crystals, yield 0.17 g (52%), m.p. 148–150 ºC. IR: υ cm^-1 ^= 3,300, 3,250 (NHNH_2_), 3,050 (CH arom.), 2,910 (CH aliph.), 1,670 (C=O). ^1^H-NMR (CDCl_3_): δ = 2.46 (s, 3H, NCH_3 _), 2.75 (t, 2H, CH_2_), 3.03 (t, 2H, CH_2_), 3.61 (s, 2H, CH_2_), 3.90 (bs, 2H, NH_2_), 5.35 (bs, 1H, NH), 7.25 (m, 3H, Ar-H), 7.56 (m, 2H, Ar-H). C_16_H_17_N_5_SO (327.41) Calcd.: C, 58.69; H, 5.23; N, 21.39; S, 9.79. Found: C, 58.45; H, 4.99; N, 21.27; S, 9.58.

*7-Methyl-2-phenylamino-5,6,7,8-tetrahydropyrido[4',3':4,5]thieno[2,3-d][1,3]thiazin-4-one* **(9)**. A mixture of the compound **5** (3.75 g, 10 mmol) and concentrated sulfuric acid (5 mL) was stirred for 6 h. The reaction mixture was then poured cautiously into cold sodium bicarbonate solution. The solid product obtained was filtered, washed with water and recrystallized from ethanol/dioxane (2:3) to give yellow crystals, yield 3.2 g (97%), m.p. 282–284 ºC. IR: υ cm^-1 ^= 3400 (NH), 1640 (C=O). ^1^H-NMR (DMSO-d_6,_): δ = 2.50 (s, 3H, NCH_3_), 2.67 (t, 2H, CH_2_), 3.03 (t, 2H, CH_2_), 4.40 (bs, 1H, NH), 7.40 (m, 2H, Ar-H), 7.67 (m, 3H, Ar-H). C_16_H_15_N_3_OS_2_ (329.44). Calcd.: C, 58.33; H, 4.59; N, 12.76; S, 19.47. Found: C, 58.13; H, 4.24; N, 12.53; S, 19.70.

*Sodium salt of 2-mercapto-7-methyl-3-phenyl-5,6,7,8-tetrahydro-3H-pyrido[4',3':4,5]thieno[2,3-d] pyrimidin-4-one* (**10**). To a solution of sodium hydroxide (0.40 g, 10 mmol) in absolute ethanol (30 mL) compound **5** (3.75 g, 10 mmol) was added and the resulting mixture was heated under reflux for 2 h. The precipitate that was formed on hot was filtered, washed with boiling ethanol and dried to give yellow powder, yield 2.8 g (80%), m.p. >300 ºC. IR: υ cm^-1 ^= 3,030 (CH arom.), 2,900 (CH aliph.), 1,640 (C=O). 

*7-Methyl-2-(methylthio)-3-phenyl-5,6,7,8-tetrahydro-3H-pyrido[4',3':4,5]thieno[2,3-d]pyrimidin-4-one* (**11**). *Method (a)*: To a solution of compound **10** (3.51 g, 10 mmol) in water (15 mL) methyl iodide (1.8 g, 13 mmol) was added under stirring at room temperature. After complete addition, the reaction mixture was stirred for 2 h further. The solid product thus formed was filtered and recrystallized from dioxane to give white crystals, yield 3.21 g (93%), m.p. 202–204 ºC. IR: υ cm^-1 ^= 2,990 (CH aliph.), 1,680 (C=O). ^1^H-NMR (CDCl_3_): δ = 2.40 (s, 3H, NCH_3_), 2.51 (s, 3H, SCH_3_), 2.74 (t, 2H, CH_2_), 3.07 (t, 2H, CH_2_), 3.78 (s, 2H, CH_2_), 7.31 (m, 3H, Ar-H), 7.55 (m, 2H, Ar-H). MS: *m/z* (rel. int.) 343 (M^+^, 80.7%), 338 (50), 300 (100), 253 (78.8), 150 (17.8), 76.83 (33), 58 (97.9). C_17_H_17_N_3_OS_2_ (343.47) Calcd.: C, 59.45; H, 4.99; N, 12.23; S, 18.67. Found: C, 59.11; H, 4.75; N, 12.17; S, 18.35. *Method (b)*: To a mixture of **7 **(3.29 g, 10 mmol) and potassium carbonate (0.5 g) in dimethylformamide (15 mL), methyl iodide (2 mL) was added and the reaction mixture was gently heated on a water bath at 60 ºC for 2 h. After cooling, the reaction mixture was poured on water, and the solid product was filtered, washed with water and recrystallized from dioxane to give white crystals, yield 0.97g (28%).

*5-Amino-1-(7-methyl-3-phenyl-5,6,7,8-tetrahydro-3H-pyrido[4',3':4,5]thieno[2,3-d]pyrimidin-4-on-2-yl)-1H-pyrazole-4-carbonitrile* (**12**). A mixture of compound **8** (0.65 g, 2 mmol) and ethoxymethylenemalononitrile (0.254 g, 2 mmol) in absolute ethanol (10 mL) was heated under reflux for 8 h. After cooling, the solvent was removed *in vacuo* and the solid residue was recrystallized from ethanol to give yellow crystals, yield 0.46 g (57%), m.p. 228–230 ºC. IR: υ cm^-1 ^= 3,300, 3,150 (NH_2_), 2,900 (CH aliph.), 2,200 (C≡N), 1,640 (C=O). ^1^H-NMR (DMSO-d_6_): δ = 2.57 (s, 3H, NCH_3_), 2.83 (t, 2H, CH_2_), 3.17 (t, 2H, CH_2_), 4.17 (s, 2H, CH_2_), 4.47 (bs, 2H, NH_2_), 7.53 (m, 6H, Ar-H + CH pyrazole). C_20_H_17_N_7_SO (403.46) Calcd.: C, 59.54; H, 4.25; N, 24.30; S, 7.95. Found: C, 59.25; H, 4.19; N, 24.28; S, 7.77.

### 3.3. General procedure for the synthesis of compounds **13–15** (from **7**) and **21–23** (from **18**) with appropriate RX

A mixture of **7 **(0.66 g, 2 mmol) or **18** (0.74 g, 2 mmol), appropriate RX (2 mmol) and sodium acetate (5 mmol for reaction with **7** or 10 mmol for reaction with **18**) in ethanol (10 mL) was heated under reflux for 3 h. After cooling, the solid product was collected by filtration, washed with water and recrystallized from appropriate solvent.

*Ethyl (7-methyl-3-phenyl-5,6,7,8-tetrahydro-3H-pyrido[4',3':4,5]thieno[2,3-d]pyrimidin-4-on-2-yl)- thioacetate* (**13**). yellow crystals, yield 0.51 g (61%), m.p. 204–206 ºC (ethanol/water 3/1). IR: υ cm^-1 ^= 3,040 (CH arom.), 2,950 (CH aliph.), 1,730 (C=O), 1,650 (C=O). ^1^H-NMR (CDCl_3_): δ = 1.30 (t, *J* = 7.1 Hz, 3H, CH_2_CH_3_), 2.53 (s, 3H, NCH_3_), 2.79 (t, 2H, CH_2_), 3.10 (t, 2H, CH_2_), 3.67 (s, 2H, CH_2_), 3.85 (s, 2H, SCH_2_), 4.23 (q, *J* = 7.1 Hz, 2H, CH_2_CH_3_), 7.33 (m, 2H, Ar-H) ; 7.55 (m, 3H, Ar-H). MS: *m/z* (rel. int.) 415 (M^+^, 70%), 372 (75), 328 (73), 285 (24), 253 (72), 122 (24), 77 (40). C_20_H_21_N_3_O_3_S_2_ (415.53) Calcd.: C, 57.81; H, 5.09; N, 10.11; S, 15.43. Found: C, 57.63; H, 5.17; N, 9.97; S, 15.35.

*7-Methyl-2-(phenacylthio)-3-phenyl-5,6,7,8-tetrahydro-3H-pyrido[4',3':4,5]thieno[2,3-d]pyrimidin-4-one* (**14**). yellow crystals, yield 0.75 g (84%), m.p. 220–222 ºC (ethanol). IR: υ cm^-1 ^= 1,690 (C=O), 1,665 (C=O). ^1^H-NMR (CDCl_3_): δ = 2.48 (s, 3H, NCH_3_), 2.74 (t, 2H, CH_2_), 3.10 (t, 2H, CH_2_), 3.60 (s, 2H, CH_2_), 4.55 (s, 2H, SCH_2_), 7.35 (m, 3H, Ar-H), 7.53 (m, 5H, Ar-H), 8.02 (m, 2H, Ar-H). C_24_H_21_N_3_O_2_S_2_ (447.57) Calcd.: C, 64.40; H, 4.37; N, 9.39; S, 14.33. Found: C, 64.05; H, 4.22; N, 9.33; S, 14.12.

*7-Methyl-2-[(2-oxopropyl)thio]-3-phenyl-5,6,7,8-tetrahydro-3H-pyrido[4',3':4,5]thieno[2,3-d] pyrimidin-4-one* (**15**). white crystals, yield: 0.52 g (68%), m.p. 215–217 ºC (ethanol). IR: υ cm^-1 ^= 2,900 (CH aliph.), 1,710 (C=O), 1,680 (C=O). ^1^H-NMR (CDCl_3_): δ = 2.50 (s, 3H, COCH_3_), 2.67 (s, 3H, NCH_3_), 2.95 (t, 2H, CH_2_), 3.23 (t, 2H, CH_2_), 3.81 (s, 2H, CH_2_), 4.03 (s, 2H, SCH_2_), 7.45 (m, 2H, Ar-H), 7.68 (m, 3H, Ar-H). C_19_H_19_N_3_O_2_S_2_ (385.51) Calcd.: C, 59.19; H, 4.97; N, 10.90; S, 16.64. Found: C, 59.03; H, 4.79; N, 10.87; S, 16.60.

*Ethyl (7-methyl-3-phenyl-6,7,8,9-tetrahydro-3H-pyrido[4',3':4,5]thieno[2,3-d][1,2,4]triazolo[1,5-*a*] pyrimidin-10-on-2-yl)thioacetate* (**21**). pale yellow crystals, yield 0.53 g (58%), m.p. 178–180 ºC (water/methanol 2/1). IR: υ cm^-1^ = 3,050 (CH arom.), 2,910 (CH aliph.), 1,730 (C=O), 1,680 (C=O). ^1^H-NMR (CDCl_3_): δ = 2.29 (t, *J* = 7.1 Hz, 3H, CH_2_-CH_3_), 2.73 (s, 3H, NCH_3_), 2.92 (t, 2H, CH_2_), 3.27 (t, 2H, CH_2_), 3.59 (s, 2H, CH_2_), 3.82 (q, *J* = 7.1 Hz, 2H, CH_2_-CH_3_), 4.27 (s, 2H, SCH_2_), 7.61 (m, 5H, Ar-H). C_21_H_21_N_5_O_3_S_2_ (455.56) Calcd.: C, 55.37; H, 4.65; N, 15.37; S, 14.08. Found: C, 55.55; H, 4.57; N, 15.56; S, 14.27.

*7-Methyl-2-phenacylthio-3-phenyl-6,7,8,9-tetrahydro-3H-pyrido[4',3':4,5]thieno[2,3-d][1,2,4]triazolo[1,5-a]pyrimidin-10-one* (**22**). yellowish crystals, yield 0.13 g (33%), m.p. 200–202 ºC (water/methanol 2/1). IR: υ cm^-1 ^= 3,050 (CH arom.), 2,910 (CH aliph.), 1,720 (C=O), 1,680 (C=O).^1^H-NMR (CDCl_3_): δ = 2.48 (s, 3H, NCH_3_), 2.72 (t, 2H, CH_2_), 3.08 (t, 2H, CH_2_), 3.63 (s, 2H, CH_2_), 4.70 (s, 2H, CH_2_), 7.34 (m, 3H, Ar-H), 7.47 (m, 5H, Ar-H), 7.79 (m, 2H, Ar-H). C_25_H_21_N_5_O_2_S_2_ (487.60) Calcd.: C, 61.58; H, 4.34; N, 14.36; S, 13.15. Found: C, 61.49; H, 4.35; N, 14.19; S, 12.99.

*7-Methyl-2-[(2-oxopropyl)thio]-3-phenyl-6,7,8,9-tetrahydro-3H-pyrido[4',3':4,5]thieno[2,3-d][1,2,4] triazolo[1,5-a]pyrimidin-10-one* (**23**). yellow crystals, yield 0.61 g (72%). m.p. 194–196 ºC (water/methanol 3/2). IR: υ cm^-1 ^= 2,910 (CH aliph.), 1,730 (C=O), 1,690 (C=O). ^1^H-NMR (CDCl_3_): δ = 2.29 (s, 3H, COCH_3_), 2.48 (s, 3H, NCH_3_), 2.74 (t, 2H, CH_2_), 3.10 (t, 2H, CH_2_), 3.64 (s, 2H, CH_2_), 4.11 (s, 2H, SCH_2_), 7.45 (m, 5H, Ar-H). C_20_H_19_N_5_O_2_S_2_ (425.53) Calcd.: C, 56.45; H, 4.50; N, 16.46; S, 15.07. Found: C, 56.25; H, 4.25; N, 16.27; S, 15.25.

*7-Methyl-2-phenylamino-3-(3-phenylthioureido)-5,6,7,8-tetrahydro-3H-pyrido[4',3':4,5]thieno[2,3-d] pyrimidine-4-one* (**16**). A mixture of **6** (1.64 g, 5 mmol) and phenyl isothiocyanate (0.68 g, 5 mmol) in absolute ethanol (10 mL) was refluxed for 4 h. After cooling, the solid precipitate obtained was filtered and recrystallized from ethanol/dioxane (2:1) as reddish crystals, yield: 2.04 g (88%), m.p. 209–210 ºC. IR: υ cm^-1 ^= 3,350, 3,300, (NH), 3,050 (CH arom.), 2,900 (CH aliph.), 1,680 (C=O), 1,220 (C=S). ^1^H-NMR (CDCl_3_): δ = 2.47 (s, 3H, NCH_3_), 3.27 (m, 6H, 3CH_2_), 7.31 (m, 10H, Ar-H), 9.25 (bs, 1H, NH), 9.55 (bs, 1H, NH), 9.94 (bs, 1H, NH). C_23_H_22_N_6_OS_2_ (462.59) Calcd.: C, 59.72; H, 4.79; N, 18.17; S, 13.86. Found: C, 59.55; H, 4.62; N, 18.35; S, 13.65.

*7-Methyl-2-phenylamino-3-phenyl-6,7,8,9-tetrahydro-3H-pyrido[4',3':4,5]thieno[2,3-d][1,2,4]triazolo [1,5-a]pyrimidin-10-one* (**17**). A solution of compound **16 **(0.463 g, 1 mmol) in ethanolic KOH (2*N*, 5 mL) was heated under reflux for 2 h. The reaction mixture was cooled and acidified with hydrochloric acid (2*N*), whereupon a solid product was separated out. This solid was filtered, washed with water and recrystallized from ethanol as buff crystals, yield 0.23 g (54%), m.p. 178–180 ºC. IR: υ cm^-1 ^= 3,200 (NH), 3,120 (CH arom.), 2,900 (CH aliph.), 1,675 (C=O).^1^H-NMR (CDCl_3_): δ 2.50 (s, 3H, NCH_3_), 2.86 (t, 2H, CH_2_), 3.19 (t, 2H, CH_2_), 6.68 (s, 2H, CH_2_), 6.92 (m, 5H, Ar-H), 7.68 (m, 5H, Ar-H), 7.92 (bs, 1H, NH). C_23_H_20_N_6_OS (428.51) Calcd.: C, 64.47; H, 4.70; N, 19.61; S, 7.48. Found: C, 64.22; H, 4.79; N, 19.50; S, 7.33.

*7-Methyl-3-phenyl-2-thioxo-1,2,6,7,8,9-hexahydro-3H-pyrido[4',3':4,5]thieno[2,3-d][1,2,4]triazolo [1,5-a]pyrimidin-10-one* (**18**). A mixture of the thienopyrimidine compound **6** (0.327 g, 1 mmol), carbon disulfide (3 mL) and potassium hydroxide (0.5 g, 9 mmol) in absolute ethanol (10 mL) was heated under reflux for 5 h. The solvent and the excess of carbon disulfide were removed *in vacuo*. The residue was treated with cold water (100 mL) and the resulting mixture was stirred then acidified with hydrochloric acid (2*N*). The precipitate thus formed was filtered and recrystallized from ethanol to give yellow crystals, yield 0.27 g (84%), m.p. 235–237 ºC. IR: υ cm^-1 ^= 3,300 (NH), 3,050 (CH arom.), 2,900 (CH aliph.), 1,670 (C=O), 1,210 (C=S). ^1^H-NMR (CDCl_3_): δ = 2.40 (s, 3H, NCH_3_), 2.70 (t, 2H, CH_2_), 2.90 (t, 2H, CH_2_), 3.50 (s, 2H, CH_2_), 5.11 (bs, 1H, NH), 7.16 (m, 5H, Ar-H). MS: *m/z* (rel. int.) 367.75 (M^-^, 36.7%), 325 (39.5), 28 (29.4), 18 (100), 17 (22). C_17_H_15_N_5_S_2_O (369.47) Calcd.: C, 55.26; H, 4.09; N, 18.96; S, 17.37. Found: C, 54.98; H, 3.94; N, 18.65; S, 17.30.

*7-Methyl-3-phenyl-6,7,8,9-tetrahydro-3H-pyrido[4',3':4,5]thieno[2,3-d][1,2,4]triazolo[1,5-a]pyrimidin-10-one* (**19**). A mixture of **6** (1.25 g, 3.8 mmol) and triethyl orthoformate (8 mL) was maintained at reflux for 4 h. After cooling, the excess of triethyl orthoformate was removed under reduced pressure. The residue obtained was recrystallized from petroleum ether/ethanol (9:2) to give white crystals, yield 0.85 g (66%), mp 243–245 ºC. IR: υ cm^-1^ = 3,050 (CH arom.), 2,910 (CH aliph.), 1,680 (C=O). 1H-NMR (DMSO-d_6_): δ = 2.19 (s, 3H, NCH_3_), 2.96 (t, 2H, CH_2_), 3.17 (t, 2H, CH_2_), 4.55 (s, 2H, CH_2_), 7.44 (m, 3H, Ar-H), 7.55 (m, 2H, Ar-H), 9.36 (s, 1H, CH triazole). C_17_H_15_N_5_SO (337.4) Calcd.: C, 60.52; H, 4.48; N, 20.76; S, 9.50. Found: C, 60.42; H, 4.22; N, 20.69; S, 9.33.

*7-Methyl-3-phenyl-1,2,6,7,8,9-hexahydro-3H-pyrido[4',3':4,5]thieno[2,3-d][1,2,4]triazolo[1,5-a]pyrimidine-2,10-dione* (**20**). A mixture of the thienopyrimidine compound **6** (0.65 g, 2 mmol) and *N*,*N`*-carbonyldimidazole (CDI) (0.64 g, 4 mmol) in dry dioxane (20 mL) was refluxed for 6 h. After cooling, the solvent was removed and the residue was triturated with cold water. The solid product was filtered, dried and recrystallized from water/ethanol (2:3) to give brown crystals, yield 0.61 g (86%), m.p. 280–282 ºC. IR: υ cm^-1 ^= 3,200 (NH), 3,050 (CH arom.), 2,910 (CH aliph.), 1,720 (C=O triazolo), 1,680 (C=O pyrimidinone). ^1^H-NMR (DMSO-d_6_): δ = 2.34 (s, 3H, NCH_3_), 2.48 (s, 2H, CH_2_), 2.87 (t, 2H, CH_2_), 3.53 (t, 2H, CH_2_), 7.43 (m, 2H, Ar-H), 7.53 (m, 3H, Ar-H), 11.95 (bs, 1H, NH). C_17_H_15_N_5_SO_2_ (353.40) Calcd.: C, 57.78; H, 4.28; N, 19.82; S, 9.07; Found: C, 57.69; H, 4.07; N, 19.91; S, 8.89.

*N-(7-methyl-2-phenylamino-5,6,7,8-tetrahydro-3H-pyrido[4',3':4,5]thieno[2,3-d]pyrimidin-4-on-3-yl)acetamide* (**24**). To a solution of **6** (0.33 g, 1 mmol), in pyridine (5 mL), acetyl chloride (0.08 mL, 1.1 mmol) was added with stirring. The reaction mixture was stirred at room temperature for 7 h further. After removal of the solvent, the solid residue was treated with cold diluted HCl, filtered, washed with water and recrystallized from ethanol/dioxane (2:3) to give fine yellow crystals, yield 0.27 g (73%), m.p. 143–145 ºC. IR: υ cm^-1 ^= 3,390 (NH), 3,050 (CH arom.), 2,910 (CH aliph.), 1,730 (C=O), 1,690 (C=O). ^1^H-NMR (CDCl_3_): δ = 2.10 (s, 3H, COCH_3_), 2.50 (s, 3H, NCH_3_), 2.73 (t, 2H, CH_2_), 3.00 (t, 2H, CH_2_), 3.57 (s, 2H, CH_2_), 4.73 (bs, 2H, NH), 7.37 (m, 2H, Ar-H); 7.47 (m, 3H, Ar-H). C_18_H_19_N_5_SO_2_ (369.44) Calcd.: C, 58.52; H, 5.18; N, 18.96; S, 8.68. Found: C, 58.35; H, 4.99; N, 18.69; S, 8.41.

*7-Methyl-2-phenylamino-3-(2-oxopropylamino)-5,6,7,8-tetrahydro-3H-pyrido[4',3':4,5]thieno[2,3-d] pyrimidin-4-one* (**25**). A mixture **6** (0.66 g, 2 mmol), and chloroacetone (0.19 mL, 2.2 mmol) in ethanol (10 mL) was heated under reflux for 3 h. After cooling, the solvent was removed *in vacuo* and the solid residue obtained was recrystallized from ethanol to give white crystals, yield 0.42 g (55%), m.p. 278–280 ºC. IR: υ cm^-1 ^= 3,300 (NH), 3,100 (CH arom.), 2,910 (CH aliph.), 1,720 (C=O), 1,680 (C=O). ^1^H-NMR (CDCl_3_): δ = 1.88 (s, 3H, COCH_3_), 2.10 (s, 3H, NCH_3_), 2.43 (t, 2H, CH_2_), 2.66 (t, 2H, CH_2_), 2.96 (s, 2H, CH_2_), 3.54 (s, 2H, CH_2_CO), 7.37 (m, 2H, Ar-H), 7.50 (m, 3H, Ar-H), 9.03 (bs, 1H, NH), 9.72 (s, 1H, NH). C_19_H_21_N_5_O_2_S (383.47) Calcd.: C, 59.51; H, 5.52; N, 18.26; S, 8.36. Found: C, 59.41; H, 5.35; N, 17.98; S, 8.39. 

*Ethyl 3-(7-methyl-2-phenylamino-5,6,7,8-tetrahydro-3H-pyrido[4',3':4,5]thieno[2,3-d]pyrimidin-4-on-3-ylimino)butanoate* (**26**). A mixture of **6** (0.82 g, 2.5 mmol) and ethyl acetoacetate (0.33 mL, 2.5 mmol), and sodium acetate (0.82 g, 10 mmol) in absolute ethanol (20 mL) was heated under reflux for 5 h. The reaction mixture was then triturated with diethyl ether (10 mL) and the precipitate thus formed was collected and recrystallized from ethanol to give buff crystals, yield 0.56 g (68%), m.p. 150–152 ºC. IR: υ cm^-1 ^= 3,350 (NH), 3,050 (CH arom.), 2,910 (CH aliph.), 1,730 (C=O), 1,680 (C=O).^1^H-NMR (CDCl_3_): δ = 1.17 (t, *J* = 7.1 Hz, 3H, CH_2_CH_3_), 1.93 (s, 3H, CH_3_), 2.48 (s, 3H, NCH_3_), 2.81 (t, 2H, CH_2_), 3.02 (t, 2H, CH_2_), 3.39 (s, 2H, CH_2_), 3.65 (s, 2H, CH_2_), 4.09 (q, *J* = 7.1 Hz, 2H, CH_2_CH_3_), 7.43 (m, 5H, Ar-H), 8.41 (bs, 1H, NH). C_22_H_25_N_5_O_3_S (439.53) Calcd.: C, 60.12; H, 5.73; N, 15.93; S, 7.30. Found: C, 60.18; H, 5.05; N, 15.95; S, 6.40.

*Ethyl 3-(7-methyl-2-phenylamino-5,6,7,8-tetrahydro-3H-pyrido[4',3':4,5]thieno[2,3-d]pyrimidin-4-on-3-ylamino)-3-oxopropanoate* (**27**). A mixture of **6** (1.64 g, 5 mmol) and diethyl malonate (5 mL) was heated under reflux for 5 h. After cooling, the excess of diethylmalonate was removed *in vacuo* and the solid residue was recrystallized from ethanol to give white platelets, yield: 1.3 g, (59%), m.p. 194–196 ºC. IR: υ cm^-1 ^= 3,300 (NH), 3,050 (CH arom.), 2,950 (CH aliph.), 1,730 (C=O), 1,690 (C=O), 1,680 (C=O). ^1^H-NMR (CDCl_3_): δ = 1.24 (t, 3H, *J* = 7.1 Hz, CH_2_CH_3_), 2.45 (s, 3H, NCH_3_), 2.70 (t, 2H, CH_2_), 3.30 (t, 2H, CH_2_), 3.33 (s, 2H, CH_2_), 3.57 (s, 2H, CH_2_), 4.16 (q, *J* = 7.1 Hz, 2H, CH_2_CH_3_), 7.36 (m, 2H, Ar-H), 7.52 (m, 3H, Ar-H), 8.98 (bs, 1H, NH), 10.09 (bs, 1H, NH). C_21_H_23_N_5_O_4_S (441.51) Calcd.: C, 57.13; H, 5.25; N, 15.86; S, 7.26. Found: C, 57.05; H, 4.98; N, 15.65; S, 6.99.

*Ethyl 4-amino-9-methyl-12-oxo-5-phenyl-8,9,10,11-tetrahydro-5H-pyrido[4'',3'':4',5']thieno[2',3':4,5] pyrimido[1,2-b][1,2,4]triazepine-3-carboxylate* (**28**). A mixture of **6 **(0.33 g, 1 mmol) and ethyl ethoxymethylenecyanoacetate (0.17 g, 1 mmol) in absolute ethanol (10 mL) was heated under reflux for 8 h. After cooling, the solvent was removed under reduced pressure and the solid residue obtained was recrystallized from ethanol/dioxane (3:1) as yellow crystals, yield: 0.39 g (87%), m.p. 214–217 ºC. IR: υ cm^-1 ^= 3,400, 3,290 (NH_2_), 3,100 (CH arom.), 2,910 (CH aliph.), 1,680 (C=O), 1,630 (C=O). ^1^H-NMR (DMSO-d_6_): δ = 1.14 (t, 3H, *J* = 7.1 Hz, CH_2_CH_3_), 2.44 (s, 3H, NCH_3_), 2.67 (t, 2H, CH_2_), 3.02 (t, 2H, CH_2_), 3.64 (s, 2H, CH_2_) 4.09 (q, 2H, *J* = 7.1 Hz, CH_2_CH_3_), 6.08 (bs, 2H, NH_2_), 7.26 (m, 5H, Ar-H), 8.29 (s, 1H, CH triazepine). MS: *m/z* (rel. int.) 450 (M^+^, 87%), 408 (22), 407 (40), 217.91 (100), 361 (29), 337 (85), 336 (45), 294 (98), 293 (19), 267 (34), 254 (13), 253 (55), 163 (13), 122 (17), 94 (13), 77 (34.6), 64 (11), 58 (27), 50.97 (13), 52 (19), 51 (16). C_22_H_22_N_6_O_3_S (450.51). Calcd.: C, 58.65; H, 4.92; N, 18.55; S, 7.12. Found: C, 58.48; H, 4.95; N, 18.40; S, 6.99.

*4-Amino-9-methyl-12-oxo-5-phenyl-8,9,10,11-tetrahydro-5H-pyrido[4'',3'':4',5'] thieno[2',3':4,5]pyrimido[1,2-b][1,2,4]triazepin-3-carbonitrile* (**29**). A mixture of **6** (0.33 g, 1 mmol) and ethoxymethylenemalononitrile (0.13 g, 1 mmol) in absolute ethanol (10 mL) was heated under reflux for 4 h. After cooling, the solvent was removed *in vacuo* and the solid residue was recrystallized from ethanol/dioxane (2:1) to give reddish crystals, yield: 0.33 g (82%), m.p. 214–216 ºC. IR: υ cm^-1 ^= 3,400,3,300 (NH_2_), 3,100 (CH arom.), 2,910 (CH aliph.), 2,210 (CN), 1,680 (C=O). ^1^H-NMR (CDCl_3_): δ = 2.75 (s, 3H, NCH_3_), 2.86 (t, 2H, CH_2_), 3.12 (t, 2H, CH_2_), 3.24 (s, 2H, CH_2_), 5.60 (bs, 2H, NH_2_), 7.35 (m, 5H, Ar-H), 8.59 (s, 1H, CH triazepine). C_20_H_17_N_7_OS (403.46). Calcd.: C, 59.54; H, 4.25; N, 24.30; S, 7.95. Found: C, 59.35; H, 4.15; N, 24.03; S, 7.88.

*5-Phenyl-2,4,9-trimethyl-8,9,10,11-tetrahydro-5H-pyrido[4'',3'':4',5']thieno[2',3':4,5]pyrimido[1,2-b] [1,2,4]triazepin-12-one* (**30**). Compound **6** (0.33 g, 1 mmol) in excess acetyl acetone (6 mL) was heated under reflux for 4 h, then the reaction mixture was concentrated and left to cool. The solid precipitate was triturated with ethanol, filtered and recrystallized from isopropanol to give buff crystals, yield 0.24 g (61%), m.p. 196–198 ºC. IR: υ cm^-1 ^= 2,900 (CH aliph.), 1,690 (C=O). ^1^H-NMR (CDCl_3_): δ = 2.11 (s, 3H, CH_3_), 2.31 (s, 3H, CH_3_), 2.51 (s, 3H, NCH_3_), 2.90 (t, 2H, CH_2_), 3.21 (t, 2H, CH_2_), 3.71 (s, 2H, CH_2_), 5.81 (s, 1H, CH triazepine), 7.26 (m, 5H, Ar-H). C_21_H_21_N_5_OS (391.49) Calcd.: C, 64.43; H, 5.41; N, 17.89; S, 8.19. Found: C, 64.25; H, 5.35; N, 17.82; S, 8.15. 

## 4. Conclusions

New pyrido[4',3':4,5]thieno[2,3-*d*]pyrimidines were obtained using ethyl 2-amino-6-methyl-4,5,6,7-tetrahydrothieno[2,3-*c*]pyridine-3-carboxylate (1) as starting material. The reaction of 1 with phenylisothiocyanate in boiling ethanol afforded the thiourea derivative 5 as the key intermediate which was reacted under different cyclization reaction conditions to form different pyridothienopyrimidine derivatives. Moreover, the cyclization reactions of the intermediate 6 led to pyrido[4',3':4,5]thieno[2,3-*d*]triazolo[1,5-*a*]pyrimidine or pyrido[4'',3'':4',5']thieno[2',3':4,5]pyrimido- [1,2-*b*][1,2,4]triazepine derivatives. In continuation of our program directed toward the preparation of new bioactive compounds as anti-infectious agents [27,28,29,30,31], the pharmacological evaluation of these synthesized compounds is under active investigation in this area.
